# Oviposition preferences for ethanol depend on spatial arrangement and differ dramatically among closely related *Drosophila* species

**DOI:** 10.1242/bio.019380

**Published:** 2016-09-30

**Authors:** Matt Sumethasorn, Thomas L. Turner

**Affiliations:** Ecological Biology, 9620 UC Santa Barbara, Santa Barbara, CA 93106-00

**Keywords:** Behavior, Evolution, Decision making

## Abstract

Recent work on the model fly *Drosophila melanogaster* has reported inconsistencies in their preference for laying eggs on intermediate concentrations of ethanol. In this study, we resolve this discrepancy by showing that this species strongly prefers ovipositing on ethanol when it is close to a non-ethanol substrate, but strongly avoids ethanol when options are farther apart. We also show fluidity of these behaviors among other *Drosophila* species: *D. melanogaster* is more responsive to ethanol than close relatives in that it prefers ethanol more than other species in the close-proximity case, but avoids ethanol more than other species in the distant case. In the close-proximity scenario, the more ethanol-tolerant species generally prefer ethanol more, with the exception of the island endemic *D. santomea*. This species has the lowest tolerance in the clade, but behaves like *D. melanogaster*. We speculate that this could be an adaptation to protect eggs from parasites or predators such as parasitoid wasps, as larvae migrate to non-toxic substrates after hatching. These natural differences among species are an excellent opportunity to study how genes and brains evolve to alter ethanol preferences, and provide an interesting model for genetic variation in preferences in other organisms, including humans.

## Abstract (precis)

**Summary:** *Drosophila* species make dramatically different egg-laying decisions depending on ethanol concentration and the distances between options. Surprisingly, we find that the species with the lowest ethanol tolerance prefers ethanol the most.

## INTRODUCTION

*Drosophila melanogaster* has become a premier model organism for the study of the connections between genes, brain, and behavior. Considerable work has utilized the courtship behavior of males as a system (Ryner et al., 1996; Spieth, 1952, 1974; Stockinger et al., 2005) because securing mates is a crucial component of fitness for *Drosophila* males (Pischedda and Rice, 2012), and males readily display these behaviors under a broad range of circumstances. Another promising behavioral paradigm is oviposition behavior in females, as it is also tightly coupled to fitness and is easy to assay in the lab (Siegal and Hartl, 1999). Because insect larvae have limited mobility relative to their parents, maternal oviposition choices are critical for fitness and have important roles in insect speciation and the evolution of insect pests (Renwick and Chew, 1994; Rundle and Nosil, 2005; Thorsteinson, 1960). Oviposition is a good system to study decisions and preferences, as egg-laying females gather and integrate multiple inputs through continual exploration and sampling before laying each egg (Yang et al., 2008). The numbers of eggs on each substrate is a clear expression of preference which is easy to quantify in a relatively high-throughput manner.

Recent notable discoveries using this oviposition as a model system for preferences have found that flies will make contrasting decisions depending on the distance between options (Joseph et al., 2009; Miller et al., 2011; Yang et al., 2008). This was first discovered by allowing flies to choose between sweet and bitter substrates (Yang et al., 2008). Though flies prefer to feed on sweet foods as adults and larvae, they prefer to oviposit on bitter substrate if sweet substrate is nearby. Flies appear to be constantly reassessing the relative positions of the available options, and will alter egg investment depending on the current distances among options (Yang et al., 2008). Similarly, although yeast is a valuable protein source for developing larvae, many genotypes of *D. melanogaster* prefer to oviposit on non-nutritious substrate if a nutritious substrate is nearby (Miller et al., 2011). As the distance between nutritious and non-nutritious substrates is increased, flies shift from strongly preferring non-nutritious substrate to strongly preferring nutritious substrate, with intermediate preferences at intermediate distances. Responses to acetic acid were found to be similar, with adult flies preferring to position themselves on substrate without acetic acid, but repeatedly venturing onto acetic acid media nearby in order to lay their eggs (Joseph et al., 2009). The adaptive significance of these behaviors remains to be determined, though reasonable hypotheses include choosing to oviposit in a place with fewer toxins, microbes, parasitoids, or large consumers, but which is near enough to the food source that larvae can find it. Regardless, initial investigation into the genes and neural circuits responsible for these behaviors illustrate the great potential to use oviposition as a model to study preferences and decision-making (Gou et al., 2014; Joseph and Heberlein, 2012; Joseph et al., 2009; Yang et al., 2008; Zhu et al., 2014).

Ethanol is particularly interesting as an oviposition cue. It has long been known that ethanol stimulates oviposition in *D. melanogaster* (Adolph, 1920), though this depends on concentration (Azanchi et al., 2013; Ogueta et al., 2010). Despite general agreement that ethanol is attractive to *D. melanogaster* up to concentrations of 10% or more, published results are sometimes contradictory (Eisses, 1997; Hougouto et al., 1982; Jaenike, 1983; McKenzie and Parsons, 1972; Ogueta et al., 2010; Richmond and Gerking, 1979; Siegal and Hartl, 1999; Zhu and Fry, 2015). A comprehensive recent experiment by Azanchi et al. clearly illustrates that ethanol is attractive at concentrations of at least up to 10% when it is close to non-ethanol substrate, at least in the *white^Ber^* strain used (Azanchi et al., 2013). Kacsoh et al., however, found that *D. melanogaster* lay eggs on 0% or 3% ethanol and avoid 6% or higher concentrations (Kacsoh et al., 2013). In this latter study the substrates were farther apart, which likely explains this discrepancy (see our results below). These two studies also began to explore the neurological basis of oviposition-related ethanol preferences. Azanchi et al. (2013) found that subsets of neurons in the dopaminergic system have opposing effects on the valence of ethanol as a cue, and Kacsoh et al. (2013) document that neuropeptide-F signaling is also involved. *D. melanogaster* and *D. simulans* were also found to dramatically alter their expressed preference in the presence of ethanol-sensitive parasitoid wasps (Kacsoh et al., 2013). This behavior was seemingly adaptive, as oviposition on ethanol (without nearby non-ethanol food) decreased survival in the absence of wasps but substantially increased offspring survival in the presence of wasps. For these and other reasons we find oviposition with respect to ethanol to be a fascinating system for the exploration of decision-making and preferences.

*D. melanogaster* has high ethanol tolerance relative to closely related species, and has likely evolved to utilize ethanol-rich substrates that other *Drosophila* species cannot (McKenzie and Parsons, 1972, 1974; Montooth et al., 2006). As such, we hypothesized that the natural genetic differences among species in the *Drosophila* genus may provide a rich substrate for understanding the genetic and neurobiological basis of ethanol-related decisions. Like humans, *D. melanogaster* is from Sub-Saharan Africa (David and Capy, 1988), and colonized the rest of the world more recently (Lachaise and Silvain, 2004). These newer ‘cosmopolitan’ populations are sometimes considered a different subspecies (‘M’ subspecies) than their Sub-Saharan relatives (‘Z’ subspecies) because of partial reproductive isolation between them (Hollocher et al., 1997; Wu et al., 1995). Most Sub-Saharan populations have lower ethanol tolerance than cosmopolitan populations (David et al., 1986; Montooth et al., 2006), and therefore might show different preferences with respect to ethanol substrates. The sister group to *D. melanogaster* contains three additional species, which are still less tolerant of ethanol: the cosmopolitan *D. simulans* and the island endemics *D. mauritiana* and *D. sechellia* (Mercot et al., 1994; Montooth et al., 2006). This last species (*D. sechellia*) is a specialist on *Morinda citrifolia* (noni) fruit (R'Kha et al., 1991), and shows an extremely low tolerance for ethanol (Mercot et al., 1994). An outgroup of the melanogaster-simulans clade includes another broadly distributed African species, *D. yakuba*, and it has a sister species (*D. santomea*) found only at high elevations on the island of São Tomé (Lachaise et al., 2000). Though there are a couple of reports concerning the oviposition preferences of *D. simulans* and *D. sechellia* (Kacsoh et al., 2013; Richmond and Gerking, 1979), little is known of the preferences of these species and nothing is known about the others in the subgroup.

Here we investigate the preferences of strains from the *D. melanogaster* subspecies, *D. simulans*, *D. mauritiana*, *D. yakuba*, and *D. santomea*, and find that there are substantial differences among them. We also find that some, but not all, of these species dramatically alter their decisions depending on the distance between options. All of these species now have reference genomes available, and many will form viable hybrids in the lab, which should facilitate future efforts to characterize how evolutionary changes in genes and nervous systems result in modified ethanol preferences.

## RESULTS

**Fig. 1.**
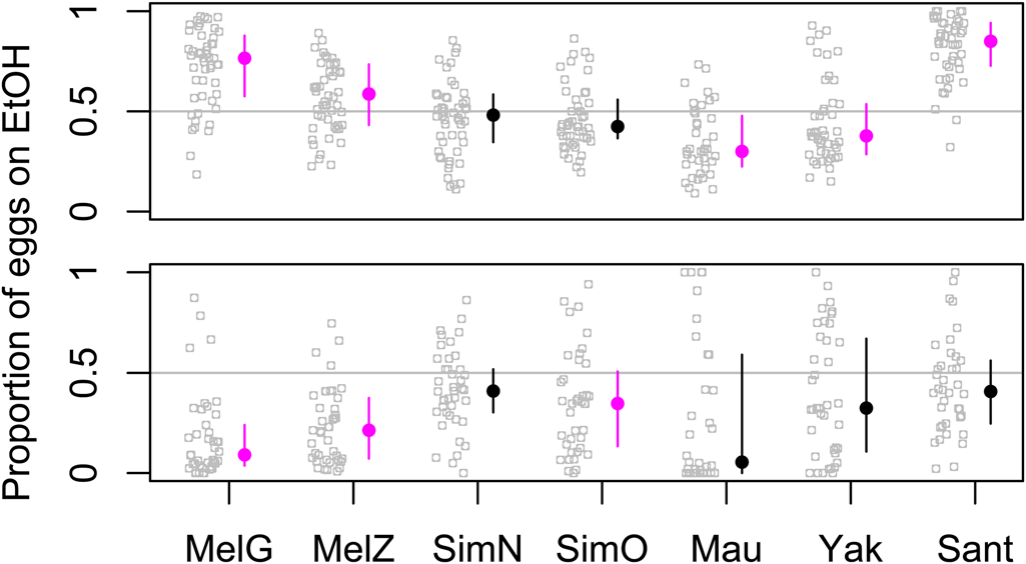
**Oviposition on 10% ethanol vs 0% ethanol.** Each strain was presented with the two substrates touching (a split-patch, top figure) or separated by 7 cm (two patches, bottom figure). Each replicate is shown as a gray circle, and the median for each strain is shown as a filled circle with a line spanning the central 50% range. Strains significantly different than random based on a binomial test, Bonferroni corrected for seven parallel tests, are shown in fuchsia.

We found major differences among strains when females were presented with the choice to oviposit on ethanol-rich or ethanol-free substrate. Pairwise comparisons of ethanol-free food versus concentrations of 6%, 10%, and 15% ethanol were conducted, but the differences among species were most pronounced at 10%, where we collected the largest data set (Fig. 1). In split-patch assays, where the two substrates are presented without a gap between them, preferences paralleled what is known about ethanol tolerance with one major exception (detailed below). Published ethanol tolerances of adult flies, from most to least tolerant, are cosmopolitan *D. melanogaster*, African *D. melanogaster*, *D. simulans*, *D. yakuba* and finally *D. mauritiana* (Mercot et al., 1994). The proportion of eggs laid on ethanol here is in the same rank order as ethanol tolerance, with the cosmopolitan strain of *D. melanogaster* laying 72% of eggs on ethanol and *D. mauritiana* laying only 35% on ethanol (Fig. 1). *D*. *santomea*, for which there are no published ethanol tolerance data, deposited 82% of its eggs on ethanol. We were surprised that females of this species preferred ethanol more strongly than *D*. *melanogaster*, as *D*. *santomea*'s sister species *D*. *yakuba* has much lower preference and tolerance.

We used the non-parametric Kruskal–Wallis test to verify that preferences varied significantly among the tested strains (*P*<2.2e-16). We also conducted a binomial test on each strain to test for significant preference for one of the options. For these tests we simply considered the proportion of replicates within a strain with greater or lesser than 50% on ethanol; these tests have lower power because a replicate with 51% of eggs on ethanol is treated the same as one with 100% of eggs on ethanol, and we Bonferroni-corrected for seven parallel tests. Nonetheless, as shown in Fig. 1, some strains clearly preferred ethanol while others significantly avoided it.

In the two-patch assays, when the same two oviposition substrates were presented to females with a 7 cm gap between them, the mean number of eggs laid on ethanol decreased for all strains, though some species like *D. simulans*, responded only slightly (Fig. 1). The biggest shifts were seen in the two strains that most strongly preferred ethanol in the spilt-patch scenario. The strain from the most ethanol-tolerant species (cosmopolitan *D. melanogaster*) now laid the fewest number of eggs on ethanol (18%). The second biggest shift in mean was seen in *D. santomea*, while its sister species *D. yakuba* changed very little. As a result, the large difference between these sister species in the split-patch scenario (Wilcoxon *P*=4.08e-11) disappeared in the two-patch case (Wilcoxon *P*=0.34). The variance among replicates within strains was higher for all strains in the two-patch case, consistent with the hypothesis that flies have a harder time comparing the available options when they are far apart. Despite this higher variance, there was still significant variation in behavior among strains in the two-patch case (Kruskal–Wallis *P*=7.38e-07).

**Fig. 2.**
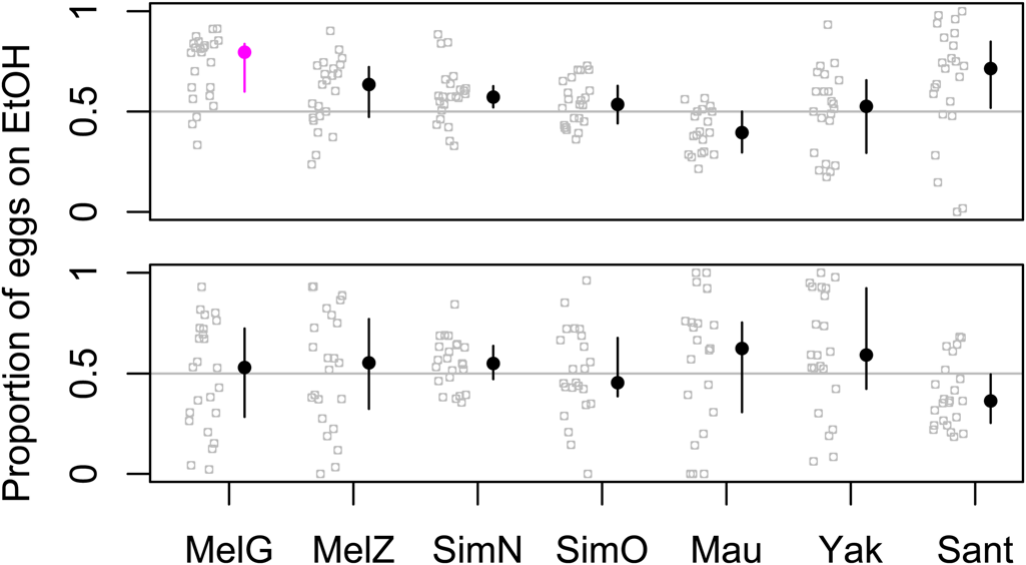
**Oviposition on 6% ethanol vs 0% ethanol.** Each strain was presented with the two substrates touching (a split-patch, top figure) or separated by 7 cm (two patches, bottom figure). Each replicate is shown as a gray circle, and the median for each strain is shown as a filled circle with a line spanning the central 50% range. Strains significantly different than random based on a binomial test, Bonferroni corrected for seven parallel tests, are shown in fuchsia.

The additional data collected comparing ethanol-free substrates to 6% ethanol were similar to that of the 10% ethanol experiment (Fig. 2). The rank order among strains was the same in the split-patch case, with the exception of cosmopolitan *D. melanogaster*, which laid slightly more eggs on ethanol (72%) than *D. santomea* (64%). Note that *D. santomea* laid more eggs on ethanol when ethanol concentrations were higher, while *D. melanogaster* did not change their behavior with increasing concentrations (mean=72% in both cases). Although differences between strains were significant in the split-plate case at 6% ethanol (Kruskal–Wallis *P*=1.51e-06), this was not the case in the two-patch assays at the same concentration (Kruskal–Wallis *P*=0.11). Just like in the 10% case, the strains that preferred ethanol most strongly in the split-patch case changed the most when these choices were placed farther apart, so that *D. santomea* and cosmopolitan *D. melanogaster* laid the fewest and the second fewest eggs on ethanol, respectively. Although sample sizes are too low for statistical significance at 15% ethanol, the rank order of strains in the split-plate case was nearly the same, with *D. melanogaster* and *D. santomea* still laying over 60% of eggs on this very high ethanol concentration, while all other strains lay below 50% (Fig. S1). When these two options were presented with a 7 cm gap between them, all strains strongly avoided the substrate with 15% ethanol (Fig. S1).

Diffusion could create a gradient in ethanol concentration when substrates are touching, and this might affect preferences (Schwartz et al., 2012). Diffusion is unlikely to be responsible for the proximity effect seen here for several reasons, including the quantitative effect of increasing distance seen in previous work (Miller et al., 2011). To ensure that the proximity effect seen here was due to distance itself rather than diffusion, we set up two additional assays. We created a split-patch assay with a piece of transparency film placed between options to eliminate diffusion. Using the cosmopolitan *D. melanogaster* strain, we found no significant differences between the preferences within the split-patch assays with or without the transparency film (Wilcoxon *P*=0.81, Fig. S2). We also collected data for the two-patch case with only 0.5 cm between options, rather than 7 cm as above. We found that *D. melanogaster* females shifted their preference significantly when options were not touching, even if they were within 0.5 cm (Wilcoxon *P*=4.4e-5). Preferences at 0.5 cm separation were intermediate to 0 cm and 7 cm, illustrating that distance has a quantitative effect on preference (Fig. S2).

### Ethanol preferences are decoupled from ethanol tolerance in *D. santomea*

**Fig. 3.**
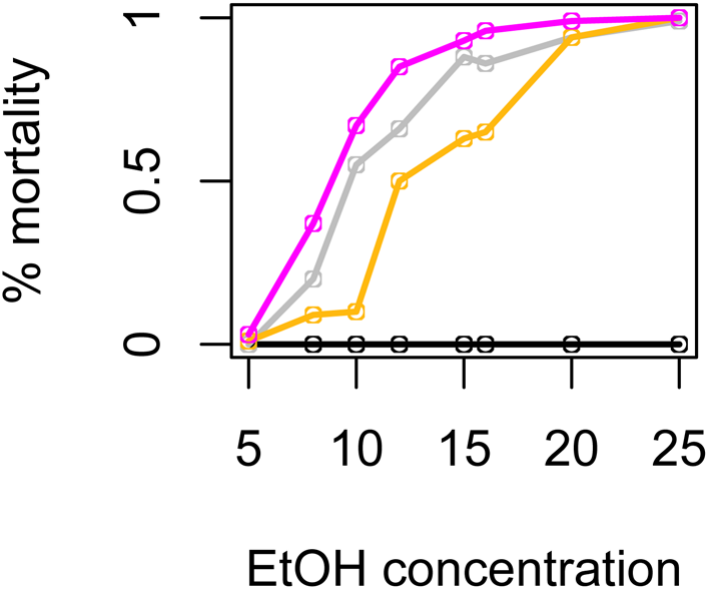
**Ethanol tolerance of adults.** The proportion of females surviving after 24 h of ethanol exposure was measured for four species: *D. santomea* (fuchsia), *D. sechellia* (gray), *D. yakuba* (gold), and cosmopolitan *D. melanogaster* (black). For simplicity, only medians are shown; sample sizes for each strain at each concentration varied from 3-16 replicate vials of ten females each. Strains were tested at concentrations of 5, 8, 10, 12, 15, 16, 20, and 25% ethanol.

*D. santomea* is the only species in our experiment with no published data on ethanol tolerance. Because this species behaved like the highly tolerant *D. melanogaster*, we predicted that it may have also evolved high ethanol tolerance. We measured ethanol tolerance by placing 10 flies in a vial with a water-sucrose solution and various concentrations of ethanol; Fig. 3 shows the proportion of flies dead after 24 h. We compared *D. santomea* to its sister species *D. yakuba*, and to cosmopolitan *D. melanogaster* (expected to have high tolerance), and finally to *D. sechellia* which is reported to have the lowest tolerance in the species group. While *D. melanogaster* suffered no mortality at any ethanol concentration tested (5%-25%), *D. santomea* suffered 93% mortality at only 10%. Indeed, *D. santomea* was similar to, and possibly even less tolerant than the sensitive species *D. sechellia*. To determine if *D. santomea* had significantly lower tolerance than *D. yakuba*, we used Wilcoxon rank-sum tests to compare mortality at each concentration. Except at the highest concentration, which is fatal to both species, and lowest concentration, these species were significantly different at all concentrations tested (*P*=0.04 at 20% EtOH, *P*≤0.006 at all other concentrations).

### *D. santomea* and *D. yakuba* differ at the species level

The big difference in preference between the sister species *D. santomea* and *D. yakuba* was surprising, as these species split only ∼400,000 years ago and still share lots of genetic variation (Llopart et al., 2002). We measured five additional genotypes per species in order to confirm that this difference is at a species level, rather than something particular to the strains used. All six of the *D. santomea* strains investigated averaged more than 50% of their eggs on ethanol, while all six of the *D. yakuba* strains averaged less than 50% of their eggs on ethanol (Fig. S3). A Wilcoxon Rank-Sum test comparing the means of the six *D. yakuba* strains to the means of the six *D. santomea* strains is significant (*P*=0.008). These data support the conclusion of a major species-level difference in preference, though there appears to also be variation among strains within a species (Fig. S3).

### *D. santomea* behavior is not inherently maladaptive

It is surprising that *D. santomea* would prefer to lay eggs on ethanol-containing substrate but have a low tolerance for ethanol as adults. One possibility is that the lab assay is unnatural enough that they are making a maladaptive choice which they would not make under natural circumstances. However, because they only exhibit this behavior when non-ethanol substrates are nearby, it is possible that it reproduces their natural behavior and is adaptive. If eggs laid on ethanol develop without problems, and larvae travel to non-ethanol substrates after hatching, there may be little to no cost in choosing ethanol substrates. Laying eggs in ethanol might then be adaptive in response to parasitoids, predators, or pathogens in nature. To begin to address this question we first determined if *D. santomea* eggs hatch equally well on ethanol and non-ethanol substrates; *D. yakuba* and cosmopolitan *D. melanogaster* were included for comparison. We saw no evidence that the ethanol substrate reduced the proportion of eggs hatched for any species (Fig. S4). For *D. santomea*, 65% and 61.5% of eggs hatched after 24 h on ethanol-free substrate and 10% ethanol, respectively (Wilcoxon *P*=0.39).

**Fig. 4.**
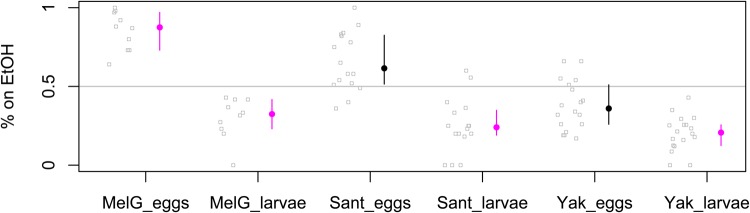
**Larval preferences for ethanol.** Each strain was presented with the two substrates touching (a split-patch, 0% vs 10% ethanol) and allowed to lay eggs. The positions of all larvae hatched after 24 h were then recorded. Each replicate is shown as a gray circle, and the median for each strain is shown as a filled circle with a line spanning the central 50% range. Strains significantly different than random based on a binomial test, Bonferroni corrected for three parallel tests, are shown in fuchsia.

We also tested whether larvae of *D. santomea* leave the ethanol substrate after hatching, and again included *D. yakuba* and *D. melanogaster* for comparison. We allowed females of each species to oviposit on split-patch assays, and then checked the positions of the larvae after 30 h (Fig. 4). As before, *D. melanogaster* and *D. santomea* laid a majority of their eggs on ethanol while *D. yakuba* did not, and variation among species in oviposition preference was again significant (Kruskal–Wallis *P*=4.52e-06). Note that the *D. santomea* data presented in this figure does not show a significant oviposition preference from ethanol unlike the data collected in previous assays. Oviposition behavior, like most behaviors, is extremely sensitive to the environment and often display differences among blocks within a strain. It is not recommended to directly compare data among different figures. Despite the caveats above, note that the point estimates of the *D. santomea* data are still >50%, which is consistent throughout all figures. After hatching, however, the larvae of all three species significantly avoided ethanol (binomial *P*<0.05 for each species after correction for multiple tests). Larvae were clearly moving away from ethanol relative to where they hatched for each species, as Wilcoxon tests comparing the oviposition preference to larval preference was significant for all three species (*P*<0.001 in all cases). In striking contrast to the differences among species in oviposition preferences, there were no differences among species in larval preferences (Kruskal–Wallis *P*=0.12).

## DISCUSSION

Though many studies have investigated how *D. melanogaster* choose oviposition substrates with respect to ethanol, our work has discovered the important role of space in modulating those preferences. We find that a cosmopolitan strain of *D. melanogaster* prefers to oviposit on substrates up to at least 15% ethanol as long as they are immediately adjacent to non-ethanol substrate. When options are separated by a gap of only a few centimeters, however, this strain strongly avoids substrates with 10% ethanol or more. This proximity effect was also seen in *D. melanogaster* from Sub-Saharan Africa. Determining the evolutionary significance of this behavior would require additional work, as our assay is designed to be simple, scalable, and replicable rather than ecologically realistic. A reasonable hypothesis might be that female flies prefer to oviposit near a larval food source rather than on it because nutritious food sources like rotting fruit can be rich with bacteria and fungi, attracting other fly larvae and also potential predators like ants and parasitoid wasps. Ethanol may offer some protection from predators and parasites with a lower ethanol tolerance (Kacsoh et al., 2013). In distances too large for larvae to easily transverse this advantage is diminished and females allocate more eggs towards sites promoting larval development. Ethanol might also serve as a cryoprotectant in temperate *D. melanogaster* populations (Zhu and Fry, 2015), though this is unlikely to be relevant in *D. santomea* which is endemic to tropical São Tomé. However, regardless of the fitness consequences, this ‘proximity effect’ has now been found for a variety of oviposition cues, and seems likely to be a general phenomenon in this species (Miller et al., 2011; Yang et al., 2008). It is important to note that we have only investigated a single strain of *D. melanogaster* from each subspecies, and there is sure to be variation in behavior among strains as we have seen for other species here and for other *D. melanogaster* oviposition behaviors (Miller et al., 2011).

In addition to clarifying the preferences of *D. melanogaster*, our results from additional species are interesting for several reasons. First, it is clear that preferences vary substantially among species. Because we raised all species in a common environment, these behavioral differences are likely to be caused by genetic differences. Naturally evolved differences are filtered by natural selection, and this may make them more likely than induced mutations to be found at key control points in the system (Stern, 2013). These genetic differences may therefore prove to be valuable tools for probing the genetic and neural substrates of decision making.

Second, we show that ethanol preference and ethanol tolerance are not completely coupled: the most and least tolerant species, *D. melanogaster* and *D. santomea*, behave in similar ways. We see a positive relationship between tolerance and preference across the other strains, which could result from pleiotropy (if the same genes affect the two traits) or intragenomic coevolution (if species with low tolerance have also evolved to dislike ethanol). In contrast, the preference differences between *D. yakuba* and *D. santomea* are not due to pleiotropic effects of tolerance genes, because the less tolerant species is the one that prefers ethanol. This makes the identity of these genes especially interesting for understanding decision making.

Finally, we see interesting differences in how species react to the distance between options. *D. santomea* and *D. melanogaster* change egg allocation dramatically with distance, but their sister taxa change very little in each case. The difference between *D. yakuba* and *D. santomea* can be conceptualized as a gene-by-environment interaction because these species do not differ when options are far apart, but do differ when they are close together (Fig. 1). The genetic basis of this divergence might therefore be informative about the mechanism underlying this proximity effect. In the long-term, we hope that combining the study of natural variation in behavior with the neurogenetic tools developed in the *D. melanogaster* model system will lead to major advances in our understanding of behavior and evolution.

## MATERIALS AND METHODS

### Fly stocks and maintenance

We used two strains of *D. melanogaster*: MelG was collected by the authors in Goleta, California in 2012; Zi237N (referred to throughout as MelZ) was collected by John Pool in the Democratic Republic of the Congo in 2010 (Lack et al., 2015). The two *D.* s*imulans* lines used were ordered from the University of California, San Diego (UCSD) Drosophila stock center: Nueva was collected in Nueva, California in 1961 (stock number 14021-251.006) and Oaxaca was collected from a mezcal factory in Rancho Zapata, Oaxaca, Mexico in 2002 (stock number 14021.051.180). The *D. yakuba* line Yaksyn2005 and *D. santomea* line Sansyn2005 were each collected by Jerry Coyne in São Tomé in 2005 (Matute et al., 2009). Sansyn2005 was made from several females from the Bom Successo field station, Democratic Republic of São Tomé and Príncipe at ∼1150 M elevation, while the Yaksyn2005 strain was made from the females collected at ∼800 M at the Pico de São Tomé, São Tomé. We used Yaksyn2005 and Santsyn2005 in all analysis, except Fig. S3 when we assessed five additional lines from each of these species all collected and provided by Daniel Ricardo Matute (University of North Carolina at Chapel Hill, NC, USA). The names of these lines are as follows, with the parenthetical numbering corresponding to Fig. S3: *D. yakuba* lines PB 1200.1 (yak1), 13.6.1 (yak2), MF 10.4 (yak3), 13.7.1 (yak4), MR 4.7 (yak5) and D. *santomea* lines CAR 1490.3 (sant1), CAR 1490.17 (sant2), 1350.14 (sant3), 1600.4 (sant4), B 1300.13 (sant5). The *D. mauritiana* stock used was from the UCSD Drosophila Stock Center and was collected at Le Reduit, Mauritius by Maria Margarita Ramos (Princeton University, NJ, USA) in 2006 (stock number 14021-041.150). We initially also included *D. sechellia* strain SynA in the experiment, constructed by Jerry Coyne (University of Chicago, IL, USA) from material collected in 1980 at Cousin Island, Seychelles, and deposited in the Drosophila Species Stock Center (https://stockcenter.ucsd.edu/info/welcome.php) by Corbin Jones (University of North Carolina at Chapel Hill, NC, USA); however this species laid very few eggs in initial assays and was not considered further. We did use this strain for comparative purposes in the ethanol tolerance assays. All fly strains were grown and maintained in 25×95 mm vials on standard cornmeal/molasses/yeast media supplemented with live yeast at 25°C on a 12 h light:12 h dark cycle.

### Oviposition behavior

Mated females were collected under CO_2_ anesthetization 13 days after eggs were laid by the previous generation and held in groups of five for 24 h. They were then introduced to the assay chambers described below, without anesthetization, using an aspirator.

Oviposition substrates consisted of 1% agar (BD #214010), 1% yeast extract (BD #212750), 1% acetic acid (Fischer #144137), 9% Trader Joe's Organic Concord Grape juice, with or without the addition of ethanol (Acros #50530496). The grape juice was first filtered through a paper coffee filter to ensure a homogeneous solution without any lingering fruit bits. Ethanol and acetic acid were added to the media after the grape/yeast/agar solution had cooled to 55°C to minimize loss of volatile fluids through evaporation. Food was then poured into the lids of 35-mm Petri plates (Falcon #351008) and allowed to congeal. For split-patch assays, razorblades were used to divide the Petri plates before pouring the media. These were removed after the media congealed. Controls for the one-patch assays were prepared by placing a small piece of transparency film in between the two substrates.

Oviposition assays were performed within 19.4 cm×18.5 cm×12.6 cm plasticware containers (Gladware #819055). Two-patch oviposition assays were made by affixing two Petri plates containing the different substrates 7 cm apart diagonally in a plasticware container. The split-patch assays had a single plate containing both options affixed to the center of the arena. A small cutout was made in the middle of the lid of the plasticware container and lined with mesh to provide venting of ethanol vapors.

Five females were aspirated into each plasticware container and allowed to oviposit undisturbed for 24 h on a 12 h light:12 h dark cycle. Data were collected in six blocks. The first four blocks had nearly equal numbers of replicates of each strain in four conditions: spilt-patch data with 6% ethanol, two-patch data with 6% ethanol, and the same two conditions with 10% ethanol. The final two blocks had nearly equal numbers of each strain in each patch type in 10% ethanol and 15% ethanol. Sample sizes for each strain and treatment combination were not precisely equal in each block because replicates with fewer than 5 total eggs were discarded.

### Ethanol tolerance

Mated females were collected under CO_2_ anesthetization 13 days after eggs were laid by the previous generation and held in groups of five for 24 h in vials. They were then introduced to the assay chambers described below without anesthetization using an aspirator. Each assay chamber was a 20 mm vial, sealed with a breathable ‘Flug’ (fly plug) closure (Genesee Scientific), containing half a fly plug saturated with a 1 ml solution of water, 0%-20% ethanol, and 3% sucrose (Fischer #BP220-1). Ten females were aspirated into each vial and left for 24 h on a 12 h light:12 h dark cycle undisturbed. Mortality of *D. melanogaster* was lower than in many published studies, which may be due to using a breathable closure. We therefore repeated the assay, but sealed the vials with parafilm: mortality was much higher, but the comparison among strains was the same. This latter, smaller dataset is included as a Fig. S5.

### Egg hatchability

Mated females were collected under CO_2_ anesthetization 13 days after eggs were laid by the previous generation and held in groups of five for 24 h in vials. Single females were then aspirated into plasticware containers and allowed to oviposit for 24 h. The female was then removed and the container placed back to remain undisturbed for an additional 24 h on a 12 h light:12 h dark cycle. Larvae and eggs were then counted at the end of the allotted time.

### Larval behavior

Mated females were collected under CO_2_ anesthetization 13 days after eggs were laid by the previous generation and held in groups of five for 24 h in vials. Five females from *D. yakuba* and *D. santomea* were aspirated into each plasticware container and allowed to oviposit for 24 h on a 12 h light:12 h dark cycle before their removal from the assay chambers. Females from *D. melanogaster* were allowed to oviposit for only 12 h before their removal to limit the total number of eggs. Eggs were counted after the allotted period. Larvae from these same eggs were then counted 30 h after the start of the assay.
